# Antitumor effect and apoptosis induction of *Alocasia cucullata* (Lour.) G. Don in human gastric cancer cells *in vitro* and *in vivo*

**DOI:** 10.1186/s12906-015-0554-2

**Published:** 2015-02-26

**Authors:** Peng Wei, Chen Zhiyu, Tang Xu, Zheng Xiangwei

**Affiliations:** Department of Medical Oncology, Fudan University Shanghai Cancer Center, Fudan University, Shanghai, China; Department of Oncology, Shanghai Medical College, Fudan University, Shanghai, China; Department of Pathology, Sichuan College of Traditional Chinese Medicine, Mianyang, China; Shanghai University of Traditional Chinese Medicine Engineering Research Center of Modern Preparation Technology of TCM, Ministry of Education, Room1107, No. 1200 Cai Lun Road, Pudong District, Shanghai, China

**Keywords:** *Alocasia cucullata*, Gastric cancer, Cell cycle arrest, Apoptosis, *In vivo*

## Abstract

**Background:**

*Alocasia cucullata* (Lour.) G. Don was applied in traditional Chinese medicine for the treatment of cancer in Chinese Southwest area. Its antitumor effect was scrutinized *in vitro* and *in vivo*. And for the first time, the mechanism of extract of *A. cucullata* (EAC) against human gastric cancer cell was well examined.

**Methods:**

To detect the most effective fraction, the antiproliferation efficacy of four fractions (namely derivatives by adding EAC to n-BuOH, petroleum ether, EtOAc and water until dissolve fully) against five cancer cell lines were screened by MTT assay. Among four fractions, the IC50s of n-BuOH fraction of EAC (EAC-B) against the five cell lines and time-dependent inhibition to gastric cancer cell line (MGC-803) were further investigated (MTT assay). *In vivo* antitumor efficacy of EAC-B was examined by MGC-803 bearing tumor nude mice. Especially, the paper focused on the relevant mechanism study of EAC-B against MGC-803 included cell cycle distribution (flow cytometry) and cyclin D1 expression (RT-PCR and western blot), apoptosis (Hoechst 33342 stain and flow cytometry), apoptosis-related protein expression (Akt, p-Akt, ERK, p-ERK, Bcl-2, Bax) by western blot, and caspase3/7 activity assay.

**Results:**

EAC-B showed its cytotoxicity against various tumor cell lines, particularly against gastric cancer cells with IC50 value of 18.8 μg/mL *in vitro*. Tumor weight was significantly reduced by EAC-B *in vivo*. In the mechanism study, EAC-B increased cell ratio at G0/G1 phase and reduced cyclin D1 expression both at protein and mRNA level on MGC-803. Chromatin condensation and apoptosis were also observed. EAC-B down-regulated p-Akt, p-ERK expression and up-regulated Bax/Bcl-2 ratio. Further, caspase 3/7 activation was enhanced as well.

**Conclusions:**

This study demonstrated that EAC-B had potent antitumor activity both *in vitro* and *in vivo*. Its mechanism is primarily via antiproliferation of G0/G1 arrest and cell pro-apoptosis, including PI-3 K/Akt pathway, ERK activity, stimulated cytochrome C release and caspase 3/7 activity accompanied with an increase of Bax/Bcl-2 ratio. EAC-B may be a potential source of novel compounds for gastric cancer treatment.

## Background

Cancer as a world-wide concern remains major threat to people health despite advances in modern medical diagnosis and treatment [1]. Gastric cancer (GC) is the fourth most common malignancy and the second most dangerous cause of cancer mortality. According to statistics in the magazine as GLOBOCAN 2008 [2], 989,600 new GC cases were recorded and 738,000 deaths occurred. GC is usually treated with adjuvant chemotherapy prior to and after surgery to prevent cancer recurrence. A meta-analysis demonstrated that 5-year overall survival increased by 6% with chemotherapy compared to surgical treatment alone [3]. A significant and continuous need for effective anticancer agents was called for. Fortunately, Paclitaxel (Taxol) is proved to be a successful drug extracted from natural product to answer the call to fill the need. The significance of healing property herbs has been highly evaluated in drug discovery. However, only 15% and 6% of these plants have been screened for phytochemical analysis and bioactivity, respectively [4].

Accumulated studies have shown that Chinese traditional herbs may reduce the side effects, prolong survival time and improve the quality of life [5]. A review showed that GC was top 3 of the reported cancers treated with traditional Chinese medicine in China [6]. For instance, “Huachansu” has shown significant effects on the improvement of leukopenia, adverse events and rate of short-term remission in the advanced or late GC patients [7].

*Alocasia cucullata* (Lour.) G. Don (Chinese taro) (AC) in the Araceae family is a fast growing and propagating herbaceous species prevalent in South China [8]. Its tuber (Chinese name “jianweiyu”) is a well-known ethnic medicine of the Zhuang nationality in China and is usually applied to reduce swelling, to detoxify and to ease pain [9,10]. For centuries in China, the interesting thing is that it has also been playing role in treatment of cancer in clinical application, including GC [11-13]. Pharmacological studies showed that an N-acetyl-D-lactosamine lectin from AC had the antiproliferation effect against human SiHa cancer cells [14], and our previous studies also proved that the 50% ethanol extract of *A. cucullata* (EAC) was found to have the most potent antiproliferation effect on five cancer cells among 50% ethanol, 95% ethanol and water extracts. EAC petroleum ether extractive fraction (EAC-PE) also showed weak antiproliferation effect on MGC-803 and 43 ingredients in this fraction were identified through GC-MS [15]. Above studies proved the antitumor activity of AC and initiated the pharmacological research of AC.

In the antitumor mechanism study, AC was reported to be effective on breast cancer [16] while *A. macrorrhiza* was reported on the hepatic cancer [17]. It was the first time for GC that the antitumor effect of EAC *in vitro* and *in vivo* were further evaluated and the possible mechanisms was elucidated by us.

In the study, the antiproliferation effect of four EAC fractions including n-BuOH fraction against five cancer cell lines was screened. HPLC demonstrated the phytochemistry difference between n-BuOH and petroleum ether fractions. Further, the inhibition to tumor growth *in vivo* was evaluated with the n-BuOH fraction of EAC (EAC-B). We also investigated the mechanisms why EAC-B inhibited the cell proliferation including apoptosis and cell cycle arrest.

## Methods

### Plant material

The tubers of *AC* were collected in Sichuan Province, China in spring, and were authenticated by Professor Baokang Huang (the School of Pharmacy, Second Military Medical University, Shanghai, China). A voucher specimen (Zheng 5470) has been deposited in the Department of Chinese Materia Medica, Shanghai University of Traditional Chinese Medicine.

### Extraction and isolation

The 50% ethanol extraction of AC was found to show stronger inhibition effect on five human tumor cells than 95% ethanol and water extraction [15]. And the extraction was processed as described previously. The fresh tubers of *AC* (90 kg) were washed, sliced, dried in the sun and then extracted with 50% ethanol three times (1:8, 1:6 and 1:6, w:v), and the extract was evaporated in vacuo to yield a residue (400 g). The residue was suspended in water, and then partitioned with petroleum ether, EtOAc and n-BuOH ordinally. The three fractions and residual of water fraction were evaporated in vacuo. The weights of the four fractions were 9 g, 170 g, 65 g and 152 g, respectively.

### Sample preparation

For HPLC analysis, 50 mg samples each were dissolved in MeOH with 1 mg/mL. The solutions were then filtered through 0.2 μm filters (Millipore Co., Bedford, MA, USA). For bioassays, 30 mg samples each were dissolved with DMSO or 70% ethanol into 30 mg/mL. And the solutions were diluted into 50 μg/mL of top dose, then with 2 fold of serial dilution. Solution stocks were stored at -20°C before use.

### Cell culture

The human cell lines, MGC-803 (gastric cancer cells), Hela (cervical cancer cells), Bel7402 (liver cancer cells), K-562 (myelogenous leukemia cells), MDA-MB-435 (breast cancer cells) were obtained from the Cell Resource Center, the Shanghai Institute of Life Sciences (Shanghai, China). All cells were cultured in DMEM (Hyclone) with 10% fetal bovine serum (Life Technology) and 1% penicillin–streptomycin in an atmosphere of 5% CO_2_ at 37°C.

### HPLC analysis

The HPLC analysis was carried out on an Agilent 1200 HPLC system (Agilent Techonologies, Santa Clara, CA, USA), combined with Prodigy ODS (2) column of 250 × 4.6 mm i.d., 5 μ (Phenomenex, Torrance,CA, USA), and guard column of 7.5 × 3.2 mm i.d.. Gradient elution was applied, using MeOH (solvent A) and water (solvent B) as mobile phase components. The flow rate was 1.0 mL/min at 25°C and injection volume was 20 μL. Gradient elution started with 5% solvent A and 95% solvent B. The elution was changed to 10% A and 90% B at 10 min, then to 50% A and 50% B at 15 min, and ended with 80% A and 20% B at 70 min. The detection wavelength was set to 205 nm (range of 196 - 450 nm).

### Cell growth inhibition assay

The *in vitro* antiproliferation efficacy of four fractions from EAC on the five cancer cell lines was investigated by MTT assay [18]. Briefly, cells were plated in 96-well plate (2000 cells/well). After plating overnight, cells were starved without serum for 24 h before treatment with 50 μg/mL of samples for 24 h. 10 μL of 0.5 mg/mL MTT was added to each well. Then read the absorbance at 492 nm. Experiments were conducted in triplicates. With the procedure above, IC_50_ were tested by 48 h treatment with various concentration of EAC-B. Additionally, EAC-B IC_50_s of 24 h, 48 h and 72 h treatment on MGC-803 were tested.

### In vivo antitumor efficacy of EAC-B on MGC-803 tumor bearing nude mice

Animal welfare and experimental procedures were in accordance with institutional guidelines for the care and use of laboratory animals and the related ethical regulations of Shanghai University of Traditional Chinese Medicine. BALB/c nude mice were subcutaneously injected at the right armpit with MGC-803 cells (2 × 10^6^/0.2 mL). All mice were sexually divided into five groups randomly: negative control (PBS), CTX control (cyclophosphamide) and three testing groups, with five mice per group. The mice in CTX control group were peritoneally injected CTX (0.05 g/kg) once a day, and the mice in drug/PBS groups were infused EAC-B intragastrically. The body weight was measured every day. On the 21^st^, the mice were killed and tumors were isolated and weighed.

### Hematoxylin and eosin (HE) staining

Ten % formalin-fixed tumor samples were dehydrated, embedded in the paraffin and sectioned into 4 μM slices. The slices were visualized with HE staining.

### Hoechst 33342 stain

MGC-803 cells were plated in 96-well plate (2000 cells/well). After plating for 24 h, cells were incubated with 50 μg/mL of EAC-B for duration of 0, 2, 8 h, then washed with PBS for twice prior to Hoechst 33342 (10 μg/mL) addition, and then incubated in dark for 20 min. The morphologic change was observed with the fluorescent microscope.

### Cell cycle analysis by flow cytometry

MGC-803 cells were plated in 6-well plates (1 × 10^5^ cells/well). After 24 h plating and then 24 h serum starvation, cells were incubated with the EAC-B for 4 h with various doses, then harvested by trypsinization, fixed with cold 70% ethanol at 4°C for 30 min and washed with PBS twice. The cell pellet was incubated in a solution containing 50 μg/mL propidium iodide (PI), 0.2 mg/mL RNase, and 0.1% Triton X-100 at room temperature in dark for 30 min. The cells were analyzed by flow cytometer (BD, FACSCalibur).

### Cell apoptosis analysis by flow cytometry

In brief, we treated MGC-803 cells with various doses of EAC-B for 4 h. Then cells were harvested, washed and incubated with the solution of Annexin V-FITC for 20 min and then PI (50 μg/mL) for 10 min. All staining operations must be carried out on ice and in dark. The cells were analyzed by flow cytometer.

### Reverse transcriptase polymerase chain reaction (RT-PCR)

MGC-803 cells were harvested after treated with 31 μg/mL of EAC-B for 4 h. Total RNA was extracted using Trizol reagent (Gibco). Equal amount (1 μg) of total RNA samples were reverse transcribed into cDNA using reverse transcription kit (Gibco) and amplified. The primer sequences are as follows [19]. Cyclin D1, forward: 5′-CGT CCA TGC GGA AGA TC-3′, reverse: 5′-CAG AGG GCA ACG AAG GT-3′ (406 bp); GAPDH, forward: 5′-CGG AGT CAA CGG ATT TGG TCG TAT-3′, reverse: 5′-AGC CTT CTC CAT GGT GGT GAA GAC-3′ (306 bp). PCR was performed as follows: denaturation at 94°C for 3 min, 40 cycles of 94°C for 30 s, 57°C for 30 s and 72°C for 2 min, followed by a final extension at 72°C for 10 min. Expression of the genes was analyzed by gel imaging system.

### Western blot assay

MGC-803 cells were treated with various concentration of EAC-B for 24 h. Cell lysates were prepared in a buffer containing 0.2% (w/v) SDS, 0.5% (v/v) Triton X-100, 0.5% (w/v) sodium deoxycholate, 1 mM PMSF, and 1% protease inhibitor cocktail. The total protein content was measured by BAC protein assay. The equal amount of protein was electrophoresed on 10% SDS-PAGE and transferred to PVDF membranes. The membranes were blocked, incubated overnight with primary antibodies at 4°C, and subsequently incubated with secondary horseradish peroxidase-conjugated goat anti-rabbit or goat anti-mouse IgG (Abcam). The following antibodies were used: Akt and p-Akt from Cell Signaling, cyclinD1, ERK, p-ERK, Bcl-2, Bax, GAPDH and β-actin from Santa Cruz. The blots were visualized using ECL detection reagents (Pierce).

### Caspase3/7 activity assay

MGC-803 cells were plated in 96-well plate (4000 cells/well). After 24 h, cells were treated with 0 to 50 μg/mL of EAC-B or culture medium (1% DMSO) for 5 h, then added the equal volume reagent of Caspase3/7 (Promega), followed with standing for 30 min. The contents of Caspase3/7 were quantified and reflected apoptosis by luminescence with microplate reader.

### Statistical analysis

Statistical software SPSS15.0 was used for statistical analysis. Data were expressed as mean ± SD. Difference between independent groups was analysed using the Student’s *t*-test.

## Results

### HPLC profile

**Figure 1:**
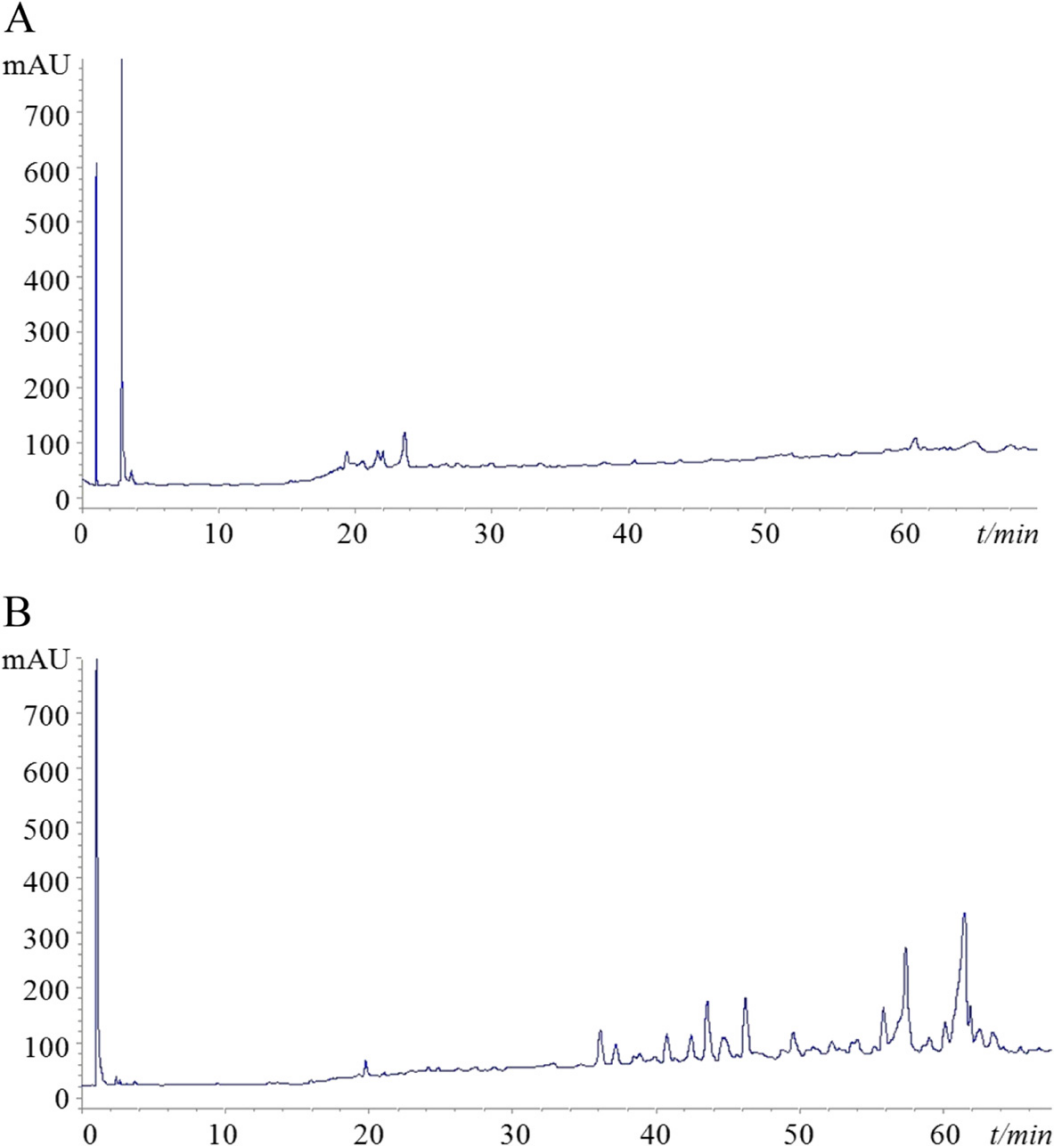
**HPLC-UV profiles of fractions partitioned from the EAC. (A)** EAC-B; **(B)** EAC-PE. (UV λ = 205 nm).

To distinguish and deepen our previous study [13,15], the HPLC chromatograms demonstrated the phytochemistry difference between EAC-B and EAC-PE. As shown in Figure 1, we detected five major peaks in EAC-B HPLC chromatograms, while eleven major peaks were confirmed in EAC-PE. The retention times were also different (EAC-B: 1.2, 2.5, 18.6, 21.9, 23.9 min; EAC-PE: 1.5, 20.2, 36.1, 37.2, 41.3, 43.1, 46.2, 49.3, 56.2, 57.5, 61.8 min). The HPLC chromatograms demonstrated the phytochemistry difference between EAC-B and EAC-PE.

### Antiproliferation activity of EAC-B in human cell lines

**Figure 2:**
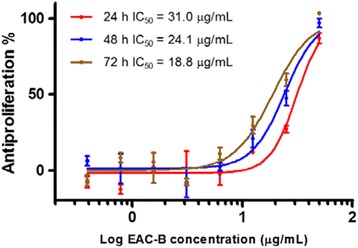
**Cytotoxic effect of EAC-B on MGC-803 in time-dependent manner by MTT assay.** MGC-803 was treated with various doses of EAC-B for 24, 48, or 72 h. *n* = 3, mean ± SD.

Four fractions of EAC were prepared and their growth inhibition activity at high concentration was identified using MTT assay against a panel of human cancer cell lines (Table 1). EAC-B showed more than 50% antiproliferative activity against four cell lines at 50 μg/mL except Hela cell. The EAC-B presented the antiproliferation on four cancer cell lines dose-dependently with 48 h treatment. MGC-803 showed the highest susceptibility to the EAC-B with the IC_50_ value of 24.1 μg/mL. Furthermore, EAC-B showed inhibition effect on gastric carcinoma in time-dependent manner (Figure 2) with the most potent IC_50_ (18.8 μg/mL) after 72 h treatment.

**Table 1:** **Inhibition effect of different fraction of EAC on 5 cancer cell lines**

**Origin**	**Single dose (50 μg/mL) inhibition at 24 h (%)**				**IC**_**50**_ **of EAC-B at 48 h (μg/mL)**
	**EAC-PE**	**EAC-B**	EAC-E*	EAC-W**	
Gastric carcinoma (MGC-803)	21.3 ± 1.3	87.0 ± 6.2	16.0 ± 2.0	12.1 ± 1.7	24.1 ± 3.7
Breast carcinoma (MDA-MB-435)	19.9 ± 3.9	76.0 ± 0.4	15.4 ± 3.7	10.2 ± 3.0	26.0 ± 2.7
Myelogenous leukemia carcinoma (K-562)	30.3 ± 0.3	73.9 ± 0.3	22.5 ± 3.8	17.3 ± 3.6	27.9 ± 7.8
Liver carcinoma (Bel7402)	18.3 ± 1.1	65.9 ± 5.1	15.8 ± 3.6	3.6 ± 3.1	39.9 ± 6.1
Cervical carcinoma (Hela)	11.6 ± 4.9	19.3 ± 1.3	13.5 ± 0.7	2.6 ± 1.5	>50

*EAC-E: the EtOAc fraction of EAC; **EAC-W: the water fraction of EAC.

### Reduced tumor growth and increased necrosis in EAC-B treated mice

**Figure 3:**
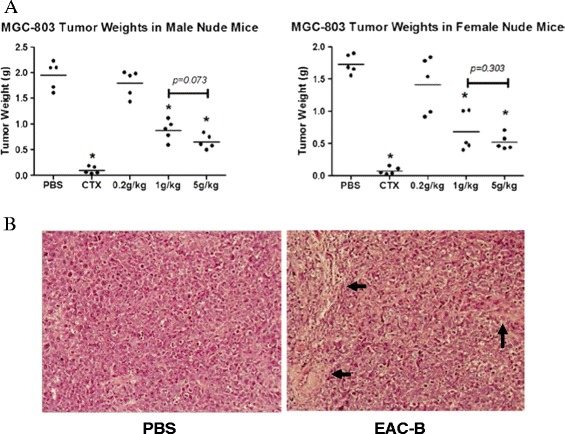
**Inhibited effect of EAC-B to tumor growth** ***in vivo*** **. (A)** BALB/c nude mice were sacrificed at the 21^st^ days after subcutaneous injection of MGC-803 cells (2 × 10^6^/mouse) followed by treatment. The treatment included of PBS (control), CTX (cyclophosphamide, peritoneal injection), EAC-B of 0.2, 1 and 5 g/kg (intragastric infusion). The scatter diagrams represented the tumor weight of each female and male animal. Significant difference were labelled with asterisk (*p* < 0.05) compared to PBS groups. **(B)** Histological change of the tumor tissue by HE staining (×20 of magnification). The loss of tumor cells (arrows) indicated necrosis (right).

EAC-B inhibited the tumor growth in a dose-dependent manner (Figure 3). 1 g/kg and 5 g/kg were effective dose with approximately 50-90% of antitumor activity. Extract treatment had better role in female mice than in male mice, especially in high dose treatment (1 g/kg and 5 g/kg). Although 5 g/kg was quite a high dose in our experiment, 5 g/kg didn’t present significant difference to 1 g/kg to the antitumor effect. Furthermore, all mice, including of mice of 5 g/kg groups, were alive until sacrifice, perhaps suggesting that the low toxicity of EAC-B. In treated mice, loss of quite amount of cells (arrows) indicated areas of necrosis by EAC-B.

### Changes of cell cycle detected by flow cytometric analysis

**Figure 4:**
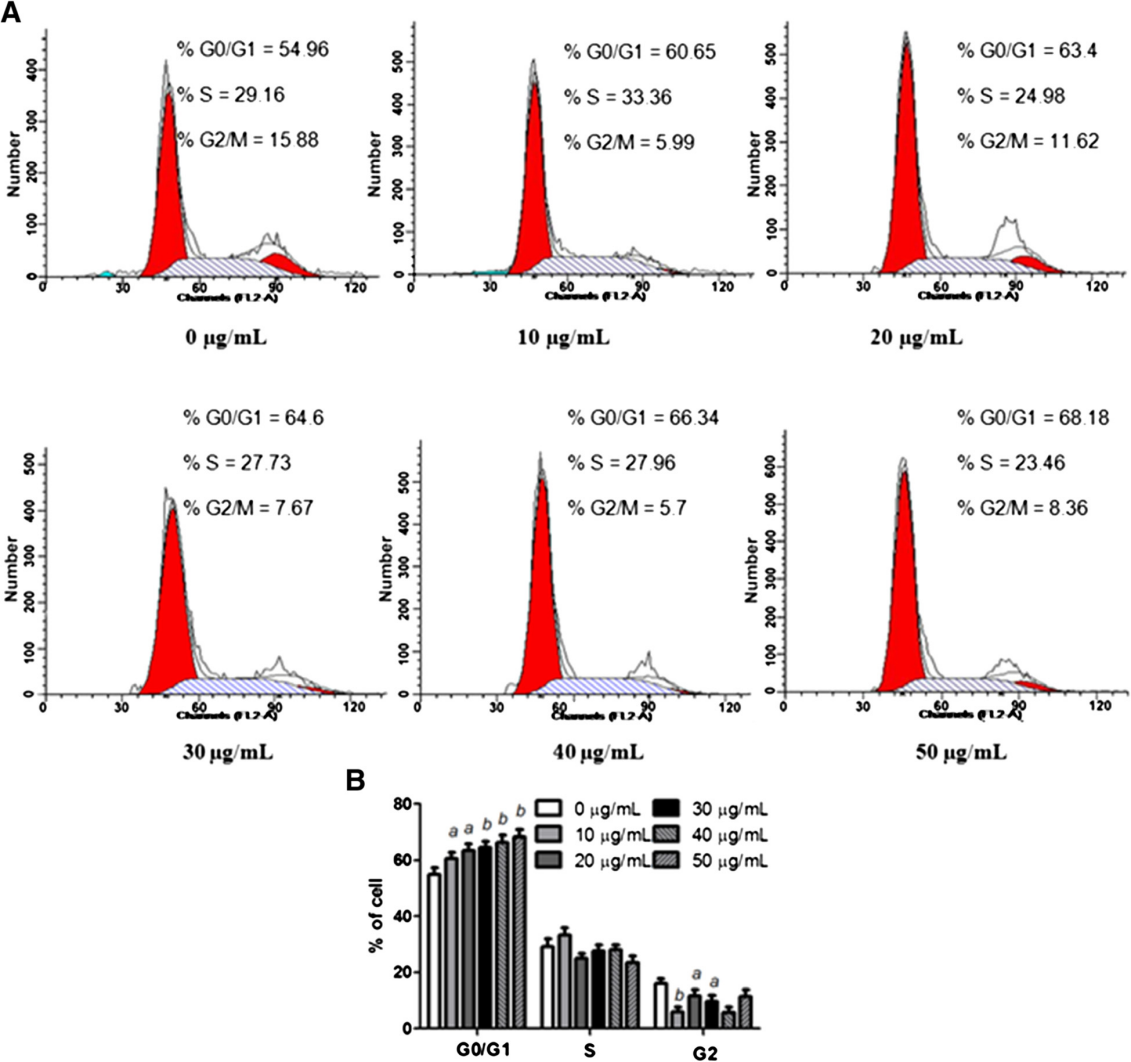
**EAC-B induced G0/G1 arrest in MGC-803 cells. (A)** Flow cytometry assay for the cell cycles of EAC-B treated MGC-803. Cells were treated with various doses of EAC-B for 4 h and then trypsinized, fixed, and stained with PI to measure cell cycle by flow cytometry. **(B)** bar graph summarized percentage of cells in every cycle. *n* = 3, mean ± SD. *a: pp* < 0.05, *b: p* < 0.01, *vs.* 0 μg/mL group.

As shown in Figure 4A and B, the cell cycle of MGC-803 cells was changed obviously. Compared to the EAC-B free group, as early as 4 h, the number of cells in the G0/G1 phase was significantly increased in a dose-dependent manner. Cell amount in the S and G2 phase did not present any trend. The progression of cell cycle was arrested at G0/G1 phase.

### Effects of EAC-B on the mRNA and protein expression of cell cycle protein (Cyclin D1)

**Figure 5:**
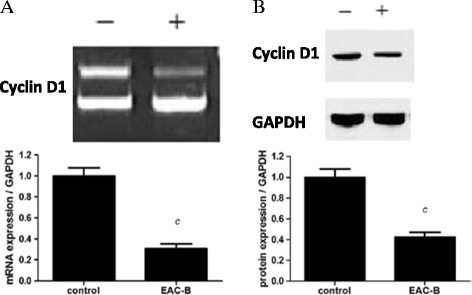
**EAC-B downregulated cell cycle related gene/protein (Cyclin D1) expression.** MGC-803 cells were treated with or without 31 μg/mL of EAC-B for 4 h prior to RT-PCR **(A)** and western blotting **(B)** analysis. GADPH was housekeeping control. Left: representative immunoblot; Right: densitomentric analysis. *n* = 3, mean ± SD. *c: p* < 0.001, *vs.* control group.

It was identified early on 4 h that EAC-B reduced the mRNA level of cyclin D1 (*p* < 0.001). Similarly, cyclin D1 protein level, detected by western blotting, was downregulated (*p* < 0.001) (Figure 5).

### EAC-B induced apoptosis in MGC-803 cells

**Figure 6:**
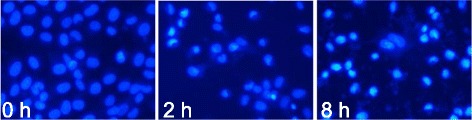
**Hoechst 33342 staining exhibited that EAC-B induced chromatin shrinking of MGC-803 cells.** Representative pictures of 3 independent experiments are shown.

In a pilot assay of Hoechst 33342, a rapid assay based on fluorescent detection of compacted chromatin in apoptotic cells, MGC-803 cells were incubated with 50 μg/mL of EAC-B by 0, 2, 8 h. After 2 h incubation, MGC-803 cells began to apoptosis. After 8 h, some cells died (Figure 6).

### Flow cytometric analysis of cell apoptosis

**Figure 7:**
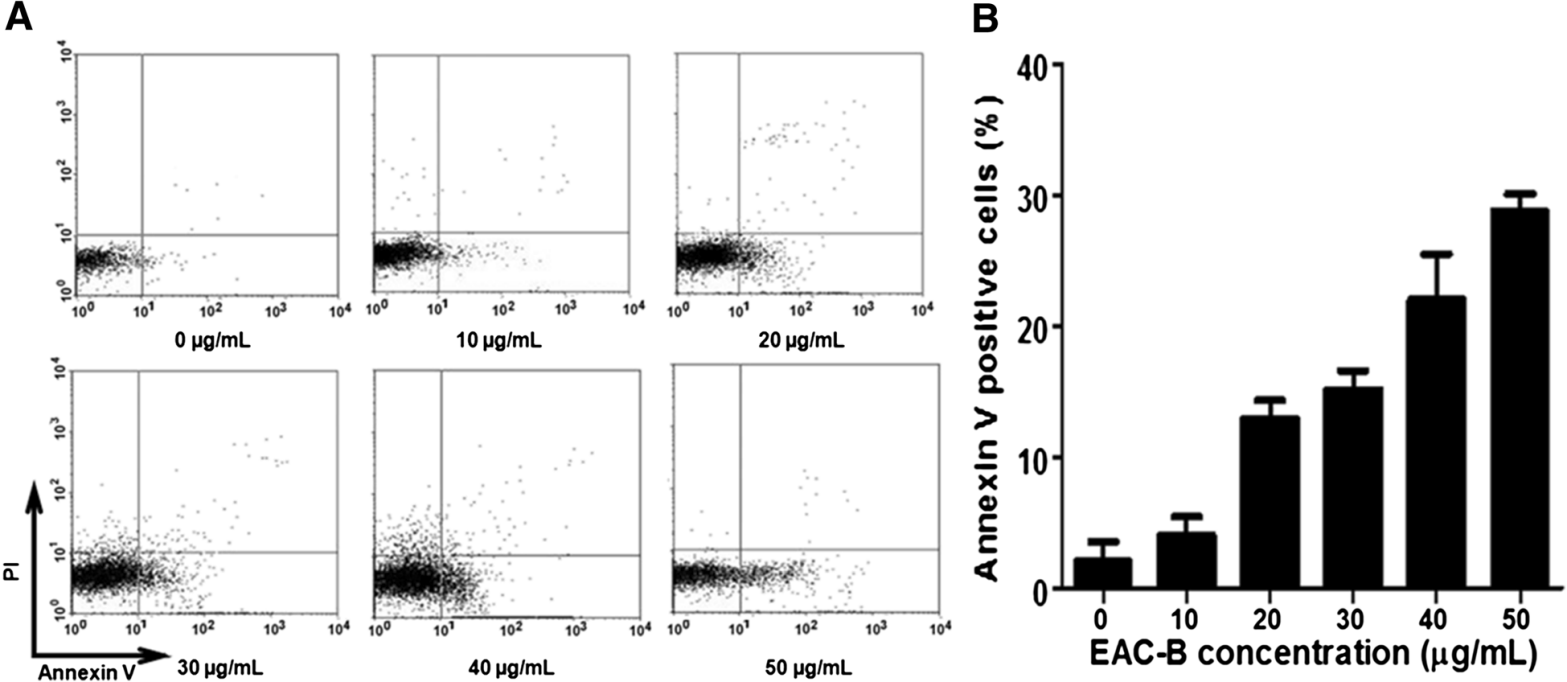
**Flow cytometric analysis of MGC-803 cells apoptosis induced by EAC-B.** MGC-803 cells were treated with different concentration of EAC-B for 4 h, and then harvested, stained with Annexin V-FITC and propidium iodide (PI). **(A)** MGC-803 cytogram from flow cytometric analysis. MGC-803 cells were treated with EAC-B at 0 (control), 10, 20, 30, 40 and 50 μg/mL. **(B)** Percentage of Annexin V positive cells. *n* = 3, mean ± SD.

Flow cytometry was used to identify and quantify the apoptosis and necrosis cells. MGC-803 cells were treated with EAC-B of 0, 10, 20, 30, 40 and 50 μg/mL. Then cells were stained with Annexin V-FITC/PI and subsequently analysed by flow cytometry. The four quadrants of the dual parameter fluorescent dot plots represented different states of the cells. The viable cells population was in the lower left quadrant (Annexin V−/PI−). The early apoptotic cells were in the lower right quadrant (Annexin V+/PI−) and the ones in late apoptosis were in the upper right quadrant (Annexin V+/PI+). As shown in Figure 7A and B, as early as 4 h, with the increasing concentration of the EAC-B, the proportion of apoptotic cells increased.

Both of the Hoechst and flow cytometry results indicated that the EAC-B may induce apoptosis in MGC-803 cells.

### EAC-B inhibited Akt and ERK in MGC-803 cells

**Figure 8:**
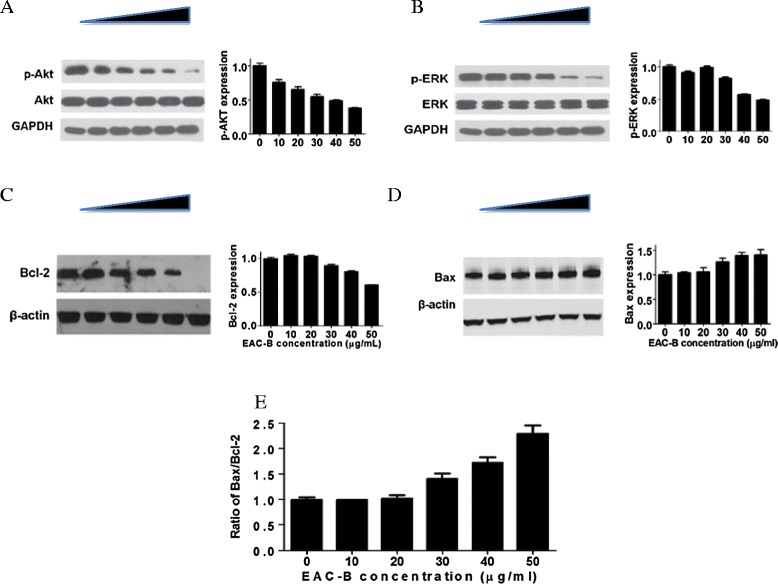
**Western blotting analysis of apoptosis regulatory proteins in MGC-803 cells after treatment with increasing concentration of EAC-B for 24 h.** Densitometry was performed for the different protein and normalized to the respective housekeeping control **(A-D)**. Bax/Bcl-2 ratio from the densitometric analysis **(E)**. *n* = 3, mean ± SD.

The biochemical mechanism by which EAC-B caused cells to enter apoptosis was explored with the PI-3 K and MAPK signaling pathway. The expression of p-Akt, Akt, p-ERK and ERK after drug treatment were measured by western blotting (Figure 8A and B). EAC-B reduced both expression of p-Akt and p-ERK by dose-dependent manner, but no effect on Akt and ERK. This means EAC-B may affect the PI-3 K and MAPK pathway in cell apoptosis.

### EAC-B increased Bax/Bcl-2 ratio in MGC-803 cells

To further investigate the effect of EAC-B on apoptosis-regulatory proteins, we also examined the expression of anti-apoptosis molecule Bcl-2 and pro-apoptosis molecule Bax in MGC-803 cells. As shown in Figure 8C and D, EAC-B significantly decreased the expression of Bcl-2 and increased the expression of Bax in a dose-dependent manner, which caused an increased ratio of Bax to Bcl-2 (Figure 8E).

### EAC-B extract activated Caspase3/7

**Figure 9:**
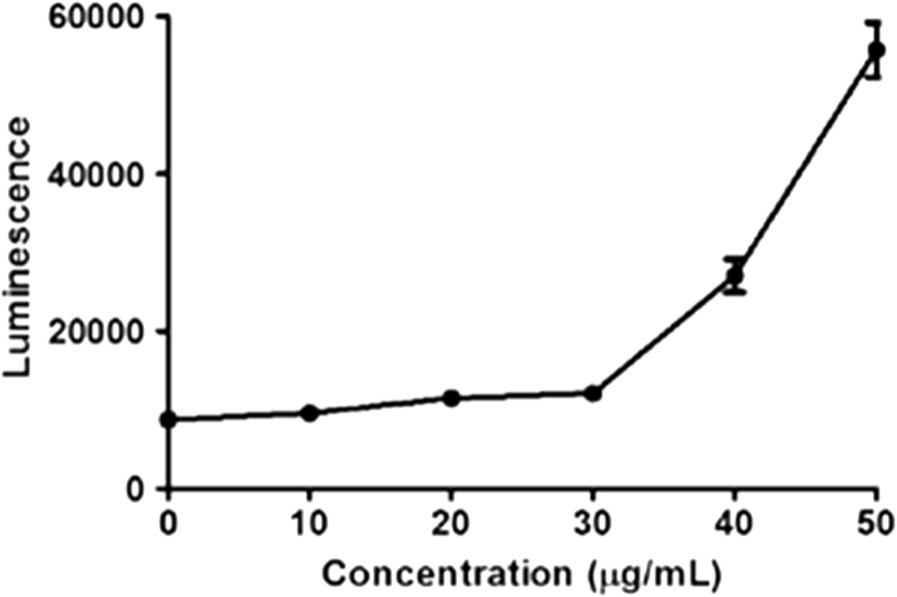
**Caspase3/7 activity in MGC-803 cells treated with 0, 10, 20, 30, 40 and 50 μg/mL of EAC-B.** The increasing caspase3/7 activity was reported as Luminescence signal. *n* = 3, mean ± SD.

Since one possible apoptotic pathway has been thought that cytochrome C released from mitochondria could trigger the caspase cascade [19], we evaluated the Caspase3/7 activity in MGC-803 cells with treatment of EAC-B. As shown in Figure 9. Caspase3/7 activity, measured by using a luminescent caspase activity assay kit, increased proportionately with EAC-B concentration of treatment in MGC-803 cells.

## Discussion

AC has been applied for treating cancer patients in traditional Chinese medicine. The antitumor effect of EAC was reported in our previous study [15], and 50% ethanol extract showed the strongest inhibition to cancer cell lines. In this paper, we further partitioned the 50% ethanol extract and investigated the antitumor effect and mechanism of EAC for the first time. The four extractive fractions were screened in terms of antiproliferation *in vitro* against various cancer cell lines. EAC-B showed the most potent antiproliferative effect on the gastric cancer cell line of MGC-803 with IC_50_ value of 18.8 μg/mL, while traditionally, AC was used in folk medicine for GC. To distinguish the component difference between EAC-B and EAC-PE [13], we demonstrated the phytochemistry by HPLC. Herein, EAC-B was scrutinized the potential and mechanism against GC *in vitro* and *in vivo*.

*In vivo*, EAC-B exerted stronger tumor inhibition on female mice than male mice, especially with high dose treatment. The gender susceptibility on GC is in accordance with the AC clinical application on breast cancer. Furthermore, compared with the highest dose (0.8 g/kg) in Fang’s report [16], we treated the model mice with higher dose treatment up to 5 g/kg. It was interesting that the mice were all alive when sacrifice, which was speculated the low toxicity of EAC-B in animal models.

Depending on the disabled cell cycle regulation, uncontrolled cell proliferation is the hallmark of cancer [20]. In cell culture, cell cycle arrest sufficient to cell senescence is a common barrier to cancer [21]. Cyclins are well-studied cell cycle regulators. Cyclin D1 is particularly required at G1 to S phase. Cyclin D1, together with its cell dependent kinase 4/6 partner, is responsible for the transition to S phase by retinoblastoma protein (Rb) phosphorylation [22]. Over-expression of Cyclin D1 oncogene can release cell from their normal cell cycle control and causes transformation to neoplasia [22]. Novel antitumor agent may derive from Cyclin D1 inhibition [23]. In our study, EAC-B inhibited the cell proliferation via G0/G1 cell cycle arrest, accompanied with a decrease of Cyclin D1 both on protein and mRNA level. Therefore, our results suggested that EAC-B suppressed MGC-803 proliferation by affecting cell cycle arrest and inhibiting Cyclin D1.

In this study, nucleus shrinking, one of morphologic alterations of apoptosis [24] was detected by Hoechst staining. Apoptotic cells ratio was increased in a dose-dependent manner by EAC-B by flow cytometry. These suggested that the mechanism of tumor inhibition by EAC-B was associated with cell apoptosis. MTT is an index of mitochondrial viability because it is reduced by metabolically active mitochondria [25]. Our results indicated that EAC-B exerted a decrease in MGC-803 cells mitochondrial viability with IC_50_ value of 18.8 μg/mL. The result suggested that the apoptosis pathway may be controlled by mitochondrion. The PI-3 K/Akt pathway is a significant anti-apoptotic/survival signaling pathway in cancer cells. Its role in the regulation of mitochondrial functions has drawn more attentions [26]. Akt, a proto-oncogene, is a key protein in this pathway. The phosphorylation of Akt leads to its activation [24]. Bcl-2 and Bax, the anti-apoptosis protein and pro-apoptosis protein, act as the downstream regulators of Akt and activated Akt, following with regulating mitochondrial outer membrane permeabilization that favors the release different apoptogenic factors, such as cytochrome C [27,28], and hence to trigger apoptotic caspase activation, including caspase 3/7. Activated Akt phosphorylates BAD, dissociates BAD from Bcl-2 or Bcl-xL and thus suppresses apoptosis [29]. Phosphorylation of Bax by Akt or activated Akt disables its translocation to the mitochondrial membrane and hence decreases permeabilization. The ratio of Bax/Bcl-2 determines the susceptibility of MGC-803 to apoptosis [30,31]. In this study, p-AKT was significantly decreased by EAC-B in a dose-dependent manner, while EAC-B treatment didn’t change AKT expression. We also found that the expression of Bcl-2 protein decreased and the expression of Bax protein increased which led to an increase of the Bax/Bcl-2 ratio. The present results showed that EAC-B exhibited antitumor by stimulating apoptosis.

A growing number of studies have confirmed the implantation of the Ras/Raf/ERK signaling pathway in the induction of cell apoptosis *in vitro* and *in vivo* [32]. ERK activity could also promote cytochrome C release by modulating Bcl-2 family protein expression. Furthermore, MEK/ERK activity has been associated with the upregulation of pro-apoptotic members of the Bcl-2 family, and the downregulation of anti-apoptotic members, such as Bcl-2 and Bcl-xL.

EAC-B treated MGC-803 cells may induce the apoptosis by inhibition of p-Akt and p-ERK, following with involvement of Bcl-2, Bax, and cytochrome C release and apoptosis execution by caspase3/7.

## Conclusions

In summary, our results showed that EAC-B significantly inhibited cancer cells growth *in vitro* and *in vivo*. The molecular mechanisms may involve 1) PI-3 K/Akt pathway of apoptosis; 2) ERK activity of apoptosis; 3) triggering of mitochondrial pathway; 4) connection by Bcl-2 and Bax; 5) G0/G1 cell cycle arrest. It is clinically confirmed that *Alocasia* is effective for cancer treatment proved by modern Chinese medicine and folk medicine, especially for GC and breast cancer. The research of *Alocasia* is not systematic despite efforts people devote to. Our study for BuOH fraction of extract from *A. cucullata* (one species of *Alocasia*)*,* may discover that EAC-B might be used as a potential agent for treating GC.

### Ethics statements

All the procedures were in strict accordance with the PR China legislation on the use and care of laboratory animals and with the guidelines established by Institute for Experimental Animals of Shanghai University of TCM and were approved by the university ethical committee for animal experiments.

## Abbreviations

AC: Alocasia cucullata (Lour.) G. Don (Chinese taro)
EAC: The extract of *Alocasia cucullata* (Lour.) G. Don
EAC-B: n-BuOH fraction of EAC
GC: Gastric cancer
HE: Hematoxylin and eosin
PI: Propidium iodide
EAC-PE: Petroleum ether fraction of EAC
